# Secondary Gerstmann syndrome, a case report

**DOI:** 10.1192/j.eurpsy.2022.1215

**Published:** 2022-09-01

**Authors:** A.L. Orozco Aguirre, M. Marín Valdovino, M.A. Ruíz Santos, A.M. González Meléndez

**Affiliations:** Hospital San Juan De Dios, Psiquiatria, Zapopan, Mexico

**Keywords:** neuroanatomy, breast cancer, Gerstmann syndrome, Neuropsychiatry

## Abstract

**Introduction:**

Gerstmann syndrome is a rare neurological disorder that consist primarily of 4 neuropsychological signs that include acalculia (impairment in performing calcultaions), digital agnosia (dificulty discriminating their own fingers), agraphia (impairment or dificulty to write by hand); and left-right disorientation (impairment of distinguishing left from right.

**Objectives:**

Presentation of a case report of a patient with Gertsmann syndrome secondary to breast cancer metastasis.

**Methods:**

We analyze the case of a 79 years-old female with a history of breast cancer in remission, with a severe depressive episode of 8 months of evolution, dysphoria, apathy, decrease in the ability to carry out basic activities of daily life, acute personality changes and sleep disruption. 15 days previous to the first examination the patient suffers gait disturbances, falling from her own height, memory impairment, suicide ideation and nomination aphasia.

**Results:**

At the examination we encounter digital agnosia, acalculia, agraphia, right-left disorientation, right hemiparesis. MRI are taken founding 3 tumor lesions in the left and right frontal lobe, 2 solid lesions with a necrotic appearance in the right parietal lobe, one of them in the angular gyrus of the parietal cortex. CT scan found a solid tumor-like lesion in the left pulmonary apex. CA-125 antigen 429.5 U/mL. She was sent to continue her treatment with oncology, receiving radiotherapy.

**Conclusions:**

The psychiatric abnormalities secondary to Gerstmann syndrome make the relatives of this patient seek psychiatric care, requiring multidisciplinary work to reach an accurate diagnosis. Gerstmann syndrome is a rare neurological condition that can mimic lots of other clinical pictures.

**Disclosure:**

No significant relationships.

